# ‘Even though you hate everything that's going on, you know they are safer at home’: The role of Aboriginal and Torres Strait Islander families in methamphetamine use harm reduction and their own support needs

**DOI:** 10.1111/dar.13481

**Published:** 2022-05-31

**Authors:** Sandra Gendera, Carla Treloar, Rachel Reilly, Katherine M. Conigrave, Julia Butt, Yvette Roe, James Ward

**Affiliations:** ^1^ Social Policy Research Centre UNSW Sydney Sydney Australia; ^2^ Centre for Social Research in Health and Social Policy Research Centre UNSW Sydney Sydney Australia; ^3^ Aboriginal Health Equity Theme South Australian Health and Medical Research Institute Adelaide Australia; ^4^ Central Clinical School, Sydney Medical School The University of Sydney Sydney Australia; ^5^ National Drug Research Institute Curtin University Perth Australia; ^6^ Psychology and Criminology, School of Arts and Humanities Edith Cowan University Perth Australia; ^7^ College of Nursing & Midwifery Charles Darwin University Brisbane Australia; ^8^ Poche Centre for Indigenous Health, School of Public Health The University of Queensland Brisbane Australia

**Keywords:** harm reduction, methamphetamine use, Aboriginal and Torres Strait Islander, First Nations people, family support need

## Abstract

**Introduction:**

First Nations people who use methamphetamine are overrepresented in regional and remote Australia and more likely to turn to family for support. This can place strain on families. The support needs of family members of individuals using methamphetamine are poorly understood.

**Methods:**

We conducted 19 focus groups and seven interviews with mostly First Nations community, family members and service providers. In total, 147 participants across six sites participated as part of a larger study investigating First Nations perspectives of how to address methamphetamine use and associated harms. We applied a social and emotional wellbeing framework to examine support needs and role of family in mitigating methamphetamine harms.

**Results:**

Findings highlighted the importance of families in providing support to people using methamphetamine and in reducing associated harms, often without external support. The support provided encompassed practical, social, emotional, financial, access to services and maintaining cultural connection. Providing support took a toll on family and negatively impacted their own social and emotional wellbeing.

**Discussion and Conclusions:**

First Nations families play an important and under‐recognised role in reducing methamphetamine‐related harms and greater efforts are required to support them. Professional resources are needed to deal with impacts of methamphetamine on families; these should be pragmatic, accessible, targeted and culturally appropriate. Support for families and communities should be developed using the social and emotional wellbeing framework that recognises wellbeing and healing as intrinsically connected to holistic health, kinship, community, culture and ancestry, and socioeconomic and historical influences on peoples' lives.

## 1 INTRODUCTION

Over the past decade, the use of methamphetamine has remained relatively stable in Australia, while the use of crystal methamphetamine, a pure and more potent form of the drug, has become more widespread [1]. Aboriginal and Torres Strait Islander people (hereby referred to as First Nations) are 2.2 times more likely to use methamphetamines compared to non‐First Nations Australians [2], and regional and remote areas appear to be disproportionally affected [3, 4]. Recent analysis suggests great variability in methamphetamine use trends in rural and remote Australia [5]. First Nations compared to non‐First Nations are also disproportionally impacted by the associated harms of regular use [6].

Methamphetamine and other drug use among First Nations people has been linked to historic and structural disadvantages, including the intergenerational impact of colonisation, racist policies and poverty [6, 7, 8, 9]. These disadvantages reinforce and compound one another while limiting the access to health care, such as comprehensive primary care. An estimated three out of 10 First Nations people who needed to access a health provider in 2018–2019, did not access services because the services were unavailable locally, there were barriers to access, such as cost, travel distance, long wait times [10] or perceptions of stigma and discrimination when accessing services [9]. Societal, economic and health disadvantages experienced by many First Nations people [11] appear to compound the disadvantage, sense of isolation and helplessness of families and communities where methamphetamine use is widespread [7].

There is scant research on First Nations perspectives about methamphetamine use and effective interventions to address associated harms to people who use, their family groups and communities [12]. To date, most research on methamphetamine use in First Nations communities has focused on epidemiological studies of patterns of use and associated harms [4, 13, 14, 15]. A recent review of the literature into methamphetamine use in Australia highlighted ‘a lack of research into specific factors within Indigenous communities’ [5]. Few treatment and other specific interventions for First Nations people who use methamphetamine are available. Evidence suggests that primary health‐care responses, culturally based interventions and utilising strength‐based community‐embedded approaches to reduce stigma and judgement have high acceptability with First Nations people [12, 16].

In a qualitative study, MacLean *et al*. [7] explored First Nations perspectives of current and past methamphetamine users, family members and parents of users. The study found that reasons for using methamphetamine were not dissimilar to those given by the general population, including the enhancement of enjoyment and the potency of the rush, participants saying they ‘needed to belong somewhere’ and the social experience of using with others [7, p. 505]. The study's findings further highlight how historical trauma, poverty and socio‐economic marginalisation ‘impeded the capacity both of the Aboriginal community and of families to deal with members' ice use’ [7, p. 504]. Current and former users spoke about ‘shame’ attached to using, especially once they were negatively viewed by members of the community, for example, due to involvement in crime or sex work. Other users spoke about wanting to hide their using habit from health services and social workers. First Nations people interviewed in that study said that they feared the violence that some associated with regular and dependent use of methamphetamine and the threats, to harm family, expressed by dealers and organised groups [7].

**FIGURE 1:**
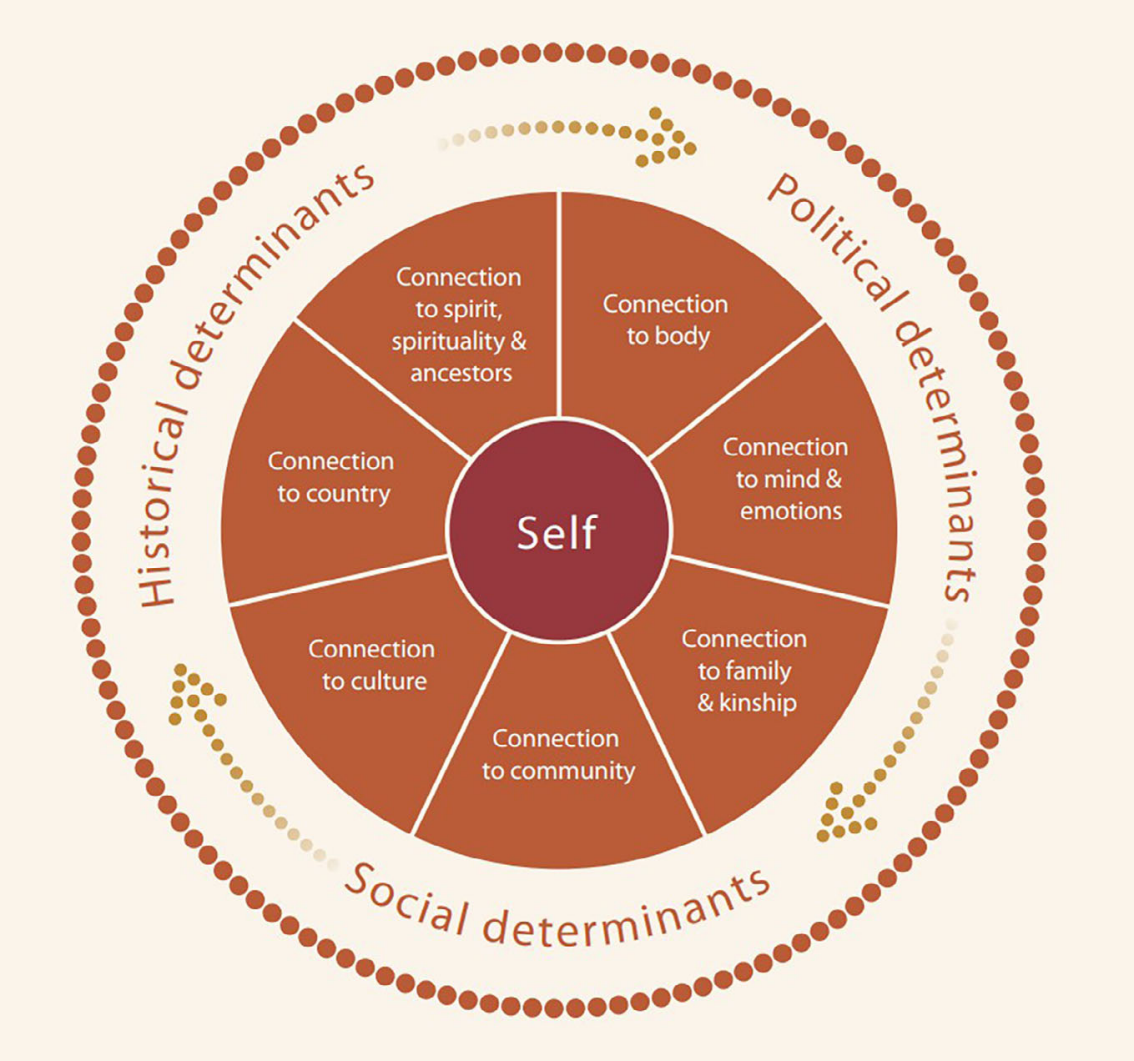
A model of social and emotional wellbeing (Reproduced with permission from Gee *et al*.,^21^ with permission.). Available from: https://www.telethonkids.org.au/globalassets/media/documents/aboriginal‐health/working‐together‐second‐edition/working‐together‐aboriginal‐and‐wellbeing‐2014.pdf.

Family and community are essential for nurturing, socialisation and survival of humans universally and are a cornerstone of First Nation societies. First Nations values, knowledge and ways of being provided the lens for understanding wellbeing and health and importantly, how this in underpinned by ‘family’, ‘extended family’ and ‘community’, alongside connection to land and culture [11, 17, 18, 19]. Appreciating the interconnectedness of family kinships is important especially as families are often the first line of support to people who use methamphetamine in a situation of limited, inaccessible or inappropriate services [20]. The Social and Emotional Wellbeing (SEWB) framework outlined by Gee *et al*. [19, 21] reflects this holistic view of health that considers the wellbeing of individuals, families and communities to be shaped by connection to health (body, mind and emotions); reliant on family, kinship and community; linked with culture, land and spirituality; and importantly, influenced by the socioeconomic and historical determinants that shape the daily lives of First Nations people [21]. These determinants include periods of history or current societal practices that remain a source of trauma and grief and contribute to the continuing greater burden of mental health and substance use problems among First Nations people [11, 19, 22, 23] (Figure 1).

The current study was conducted in the context of scant evidence on the experiences and perspectives of First Nations family, community members and people with experience of methamphetamine use to inform harm‐reduction efforts. We applied the SEWB framework to examine the role that First Nations families have in addressing methamphetamine‐related harms in the life of someone using methamphetamine. Through this lens, we describe the forms of support considered vital for First Nations family members to maintain their own SEWB. We used the SEWB framework as an analytical lens to explore three questions: (i) What types of support do family community networks provide to those who use methamphetamine?; (ii) What is the impact on families of providing this support?; and (iii) What support mechanisms do families and extended families need to effectively support someone who uses methamphetamine? While focused on families, we applied each of the domains of the SEWB framework to examine these questions in a holistic analysis.

This paper reports findings from focus groups and interviews conducted in the context of a larger study entitled ‘Novel Interventions to address Methamphetamine use in Aboriginal and Torres Strait Islander Communities’ (NIMAC: www.nimac.org.au). The NIMAC project seeks to better understand and address the needs of First Nations communities in relation to harmful methamphetamine analysing qualitative and quantitative data on patterns, determinants and consequences of use, to inform the development of prevention and treatment interventions that are strengths based and culturally appropriate.

## 2 METHODS

### 2.1 Study design and data collection

This study, conducted between 2017 and 2019, was a partnership between academic researchers and Aboriginal Community Controlled Health Services (ACCHS) in eight sites in regional (six), remote (one) and urban (one) locations in five Australian states and territories. The eight communities self‐selected to be part of the study. ACCHS are operated by local First Nations communities delivering holistic, comprehensive and culturally competent primary healthcare services. This includes incorporating alcohol and other drug (AOD) prevention and treatment, and important centres for cultural and community connection. This study is a sub‐study of a broader research program (NIMAC: www.nimac.org.au) which explored risk and protective factors of methamphetamine use in six First Nations communities (five regional and one urban locations in five Australian states and territories) from the perspectives of three groups: (i) those who have used or use methamphetamine; (ii) community members (e.g. Aboriginal elders) and family members affected by methamphetamine use; and (ii) service providers working in AOD‐related fields.

A semi‐structured discussion guide was developed with input from study investigators and Aboriginal and Torres Strait Islander community researchers. The ACCHS partners employed community researchers to coordinate participant recruitment and data collection. Community‐based researchers conducted the focus groups and interviews with support from members of the research team, or other senior researchers within their own organisations. Senior community members guided the research team to select the appropriate setting and composition of focus groups and interviewees. Yarning as a mechanism was used by community researchers in the focus groups and interviews. This approach enabled the discussion and questions to remain fluid, with an intent to create a collaborative space, equally valuing the voices, interests and experiences of the study participants and researchers [24]. Focus groups and interviews were broadly organised in three parts, directed by the particular interests of each focus group or interview: (i) risk and protective factors of methamphetamine use—thinking about circumstances at community and individual levels that may increase or decrease the vulnerability of young people to engage in harmful methamphetamine use; (ii) problems associated with methamphetamine use for families, communities and the person using; and (iii) strategies to address and prevent methamphetamine use and related harms. Focus groups took between 1.5 and 2 h, interviews around 1–1.5 h.

### 2.2 Sampling, recruitment, ethics and cultural protocols

Participant recruitment and data collection were informed by national guidelines for research with Aboriginal and Torres Strait Islander people [25] and participant feedback concerning cultural protocols. For example, we respected preferences, such as not to be in a group setting, number of participants in a focus group, gender sensitivities and advice from community cultural authorities on attendees. Study participants with experience of methamphetamine use and community members were recruited via advertisements in community organisations directing them to speak with their local community‐based researcher and through snowball sampling. Several people with known experience of use were approached in person by a community researcher. Sites were recruited by approaching senior staff in relevant organisations with information about the project. Participation in the study was voluntary. Due to the sensitive nature of the study, participants who did not want to speak in a group were offered individual interviews. Focus groups and interviews were carried out in community settings considered safe, comfortable and convenient for the study participants, including health services and community organisations. Three focus groups were carried out in residential rehabilitation services. Where available, the project was approved by the local State and Territory Aboriginal Human Research Ethics Committee (South Australia, New South Wales, Western Australia). In Victoria, approval was granted by the St Vincent's Hospital Melbourne Human Research Ethics Committee, in Queensland by the Belberry Human Research Ethics Committee and in the Northern Territory by the Human Research Ethics Committee of the Northern Territory Department of Health and Menzies School of Health Research. All data collected was owned by the ACCHS with consent provided to the research to conduct the analysis thereby supporting First Nations community data sovereignty [25].

### 2.3 Data analysis

The qualitative data were voice recorded and transcribed verbatim. Deidentified transcripts were uploaded into NVivo11 for analysis. All participants were offered the opportunity to review their transcript and provide feedback prior to analysis. Three participants requested copies of their transcripts and did not provide feedback. We applied the SEWB framework [19, 21] to examine the role of family and extended family in mitigating methamphetamine harms and their support needs. Codes were applied to the data using the SEWB framework using deductive coding. We used each dimension of the framework (connection to mind and emotions; connection to family and kinship etc.) to examine each of the three research questions regarding support provided by family members. For example, we explored the support provided by family members using each domain of the SEWB framework, including interactions between them as well as situating this analysis in their social, political and historical environments (such as the lack of services or expertise in areas outside urban areas, or racism within services or communities). Preliminary analysis was undertaken by non‐First Nations researchers (SG, CT, RR). These preliminary analyses of each dimension of the framework were circulated to the full authorship groups to ensure, in particular, that First Nations researchers closely reviewed the analysis and the application of the SEWB framework and added to the interpretation of the findings. Community‐based researchers who had been involved in data collection reviewed the interpretation for accuracy. A summary of the initial analysis, undertaken by non‐First Nations researchers (SG, CT, RR), was presented to members of the research group with a specific workshop‐style discussion with Indigenous researchers (JW, YR) to review the draft and elicit further reflection and interpretation [26].

## 3 RESULTS

Data were collected during 19 focus groups with 35 people with past or current experience of use, 50 people who identified as a community or family member, and 55 service providers, and seven individual interviews. In total, 147 participants took part in the research and, of those, the majority identified as First Nations (*n* = 118, 80%) (Table 1). Participants were 65 women and 83 men aged 16–69 years (mean 40 years). Of the 35 participants with experience of use, 20 were receiving treatment for methamphetamine dependence at the time of data collection. Most participants in the service provider group were employed in the AOD or mental health sectors as counsellors or Aboriginal health workers. Other professions represented justice support workers, police, nurses, allied health staff and youth workers.

**TABLE 1:** Focus group and interview participant gender, First Nations status and experience with methamphetamine use across all sites

	*n*	Female	First Nations
*Focus groups (n = 19)*			
Total focus group participants	140	70	113
People with experience groups	35	14	35
Community and family groups	50	14	50
Service provider groups	55	42	28
*Individual interviews (n = 7)*			
Total individual interviews	7	5	5
People with experience	4	3	2
Community and family member	2	2	2
Service provider	1	0	1
*Total participants in focus groups and interviews*	*147*	*75*	*118*

### 3.1 Types of support provided by families to people using methamphetamine

#### 3.1.1 Maintaining connection and care

Immediate and extended family members were regarded as a source of emotional strength and practical support with the goals of maintaining connection to culture and minimising the harms of use, like malnourishment or homelessness. Accounts from parents, siblings and grandparents revolved around maintaining family bonds and support (‘just be there for them’), providing a safe space and roof over the head, food and nutrition, financial assistance, such as paying for bills, private therapy and other health care needs, as well as attending to a loved ones' needs when they are experiencing withdrawal symptoms. Grandparents in the role of primary carers said that they wanted to support and raise their grandchildren because they loved them, but also, ‘doing the right thing, taking in […] grandchildren to keep with the culture’ (Grandparent, Community B. Focus Group_3). All service providers emphasised the vital role that family and extended family play in maintaining methamphetamine users' links to community, practical and wellbeing support and preventing homelessness. Participants perceived that once the links to family and community have broken down, people with problematic use were more vulnerable to detrimental outcomes.

‘[Without support from their family] *often their health just deteriorates and all their social structures*. […] *They don't eat, they don't sleep*. […] *Something to do with it is homelessness, because a lot of them do end up homeless and it is a big thing*.’ (Service provider, Community C, Focus Group 17)

#### 3.1.2 Service access and crisis support

Participants frequently reported that they did not have the knowledge or confidence to seek assistance and had insufficient information about the types of support available to assist their close family. Several participants who cared for a family member with dependent use said that they helped them access crisis support and hospital care, specialist AOD services, psychology, counselling and rehabilitation services, as well as helping them with court appearances or dealings with police. Sometimes, family members had to call upon emergency services to intervene in more critical circumstances. Some family members who were involved with general hospital, specialist and emergency services, reported mixed or negative accounts of service interactions.

‘*The hospitals need to be better educated on ice users and impact*. […] *Get them* [all hospital staff] *educated and don't treat me like shit when I come in there. Like, seriously … treat me the same way you would if it was any other sort of medical issue*.’ (Family member, Community C, Focus Group 17)

#### 3.1.3 Post‐rehabilitation support

Some parents spoke about supporting a loved one after they had left residential rehabilitation, by ‘knowing the triggers’; places, contexts and cues that can stimulate a person to think of using. One mother recalled how she organised alternative activities for her two children post rehabilitation.

‘*Because it is all about their endorphins and everything else. Releasing the endorphins, that's what the ice does, is to replace it with something else, a natural high. So, a natural high is what you are looking at, that's why I liked that idea with boxing*.’ (Family member, Community A, Interview 5)

#### 3.1.4 Families could enhance the risk of drug use

The role of family and extended family in methamphetamine use in First Nations communities was discussed as a protective factor, to mitigate and minimise the harms of drug use, support access to treatment, as described above and that these protections included all components of the SEWB Framework. However, family members holding favourable attitudes towards drugs, a family history of drug use or intergenerational use, were identified as a significant risk factor that exposed young people to methamphetamine and other drug use.

### 3.2 The impact of providing support

#### 3.2.1 Emotional, physical and psychological impact

Participants discussed their struggle to cope with feelings of guilt, fear of losing loved ones to overdose, fear of gangs and criminal activity, leading to fear for their own safety and that of other family members. Disappointment and fear were often intertwined with anger and frustration of ‘not knowing’ what do to, how to deal with the situation, how to assist someone with dependent use and concerns about further harms.

‘*All of my 50 plus years… I'm naïve when it comes to ice*. […] *I don't know how to cope with it. Other than tell them, “Don't do it. It's no good for you,” but they don't listen. I slapped my 30‐year‐old daughter. I shouldn't be slapping my 30‐year‐old daughter because she's taking ice. Yeah, I find it really hard. It breaks up the family. We had a big argument, I kicked her out of the house, “I don't want you here as long as you're doing this shit”*.’ (Family member, Community B, Focus Group 5)

‘*Even though you hate everything that's going on, but you know they are safer at home and … you hate that coming and going and the aggression, you know that complete personality change. You know, like you are walking on eggshells all the time with that and but, you know if you kick them out, they're only going to get more angry, more hurt, then the substance abuse will be more, and that's when they end up dead*.’ (Family member, Community A, Interview 5)

The majority of family members spoke of the detrimental effects that providing accommodation, along with support, love and care had on their wellbeing. Physical symptoms included insomnia, restlessness and fatigue. Participants reported deterioration of their emotional wellbeing and mental health from sleepless nights, worry, shock and trauma, and the social isolation and perceived stigma that often comes with dealing with drug use in one's family.

*‘When they disappear for weeks on end, that's the part that really hurts because you don't know where they are, what they're doing*.’ (Family member, Community F, Focus Group 18)

‘*You get worried when they talk about necking themselves and committing suicide. And you don't know whether they're going to do it or not*. […] *You can't sleep because you think you might get up and find a dead body in your house*. […] *They* [young people] *think it's just affecting them, but it's affecting everyone around them*.’ (Family member, Community C, Focus Group 17)

#### 3.2.2 The impact of judgement and stigma on seeking support

Service providers confirmed that many family members felt helpless and ‘burnt out’ but often did not reach out for support due to the judgement and criminalisation attached to drug use and addiction, fear of stigma and racism connected to help seeking for this group, negative experiences and distrust in services.

Interviewer: ‘*Why do families not seek out support or assistance?*’

Participant: ‘*I think it is the stigma attached with abuse, substance abuse. People are made to feel very low, which is wrong, which is so wrong. They don't need to be put down*.’ (Community member, Community A, Focus Group 12)

Significant social isolation was identified as a detrimental outcome for the family as a whole, partly a result of the household not seeking out professional or informal community support (from friends, Elders and neighbours), when available. Most often, however, professional and culturally appropriate support was reported to be unavailable.

### 3.3 Types of support and interventions needed for family supporters

Mirroring all components of the social and emotional wellbeing model, participants discussed the types of support family members and supporters required to maintain their own SEWB and enable them to provide support to a family member who uses.

#### 3.3.1 Education to care for someone who uses and self‐care

Appropriate support for family members was described as targeted, accessible and relevant information and education about the effects of methamphetamine use, how users can remain safe while using, signs of dependent use, and how to manage and deal with problematic use. Some participants suggested a helpline that can be accessed for confidential general advice on drug use and professional advice on how to manage more difficult behaviours.

‘*We need information around* … [about drug use and addiction] *and community support, you know. Yeah*. […] *information or building the family community members up around it, to make them a bit more resilient, stronger and have them put boundaries in place, and trying to figure out, you know, ways of keeping them safe. And not having that guilt and shame put on themselves*.’ (Family member, Community D, Focus Group 7)

Participants stated that information and education for family supporters should focus on how they can remain safe themselves and setting boundaries. Understanding and responding to one's emotions (anger, sense of helplessness, fear) or dealing with the impact of substance use on the family's functioning (managing shared finances, family discord), were some of the concrete examples raised by family members and service providers. Participants also stressed that the support for families should focus on building resilience, reducing stress and encourage connections to peers, culture and healing (see next point). Support for First Nations families should likewise include access to culturally safe counselling services for individuals and groups.

#### 3.3.2 Peer groups, cultural connection and activities, and appropriate support

Participants highlighted the need to offer a range of community‐run groups for family and community to come together, share their stories and experiences, reduce social isolation and stigma and enable individual family supporters to build their networks of social support.

‘*I think another good group too, would be, is a community group that supports family members of users because they've got some stories to tell. And we could get a family group like mothers, aunties, sisters, whatever together, the family group and say, “Hey, this is what I've been through*.”’ (Women's group, Community D, Focus Group 8)

Participants suggested that art programs, cultural activities, connection to country and spirituality should be run by First Nations organisations and within primary health initiatives that target general wellbeing rather than drug use information per se. Not singling out a particular family or group, respecting privacy and confidentiality, and embedding support in cultural knowledge, were all identified as key elements of culturally appropriate support.

#### 3.3.3 Bringing the community together

In the context of methamphetamine use and support for First Nations families and communities, participants repeatedly discussed ‘collective’ approaches and support for the ‘local community’ and this support should relate to the overarching social, political and historical determinants of SEWB. In most focus groups and interviews, participants discussed the importance for policy to address poverty, lack of work and educational opportunities in their community and the need to connect to culture and build community cohesion. Collective strategies to connect to culture, land and ancestry included bringing people together via community barbeques, art festivals, grass‐roots mentoring programs or taking young people ‘out bush’ (to country). The focus of strengths‐based, community‐embedded approaches should be on building resilience and sense of belonging of individuals, families and the community overall.

## 4 DISCUSSION

First Nations families play a central and under‐recognised role in the support of people who use methamphetamine and in reduction of drug‐related harms. The care provided by families encompassed: practical, material, financial and wellbeing support; help to access health and emergency services; social care, such as looking after grandchildren or support post rehabilitation; and lastly, maintaining a connection to culture. Often families provided support without external (professional or informal) help due to a lack of appropriate services, stigma and racism attached to accessing services for this population, and limited knowledge about how to seek help. Most study participants underlined the essential role of family in supporting people who use, however family can also present a risk factor, where young people grow up in a social environment favourable towards drug use [27].

Providing support took an immense toll on the emotional, mental, physical health, social wellbeing and community connectedness of First Nations family members. These results are similar to research with parents and family of non‐First Nations backgrounds. The stigmas attached to drug use impact on families' sense of isolation and help‐seeking and can exacerbate trauma and grief experienced by families [28, 29, 30].

In line with existing research [7, 12], our results with First Nations families and communities confirm the need for broader community based and culturally tailored interventions, taking a holistic and integrated view of the issues families and communities are dealing with. In line with the SEWB framework, overwhelmingly study participants—family and community members, people with experience of use and service providers—identified a broad range of approaches to support family carers and communities in their own needs and in their efforts to reduce the harms of methamphetamine use. Specific suggestions included: tailored resources (websites and phone helpline for supporters); professional services (primary health working in collaboration with local knowledge, counselling and outreach support); grassroots approaches that foster connection to community, mutual support and cultural connection (Aboriginal run art or cultural programs, peer groups, individual and group mentoring to break social isolation and stigma); addressing the economic and historic determinants that shape First Nations livelihoods (racism and lack of opportunities); and initiatives to engender greater community cohesion and cultural connection for individuals, families and communities.

Two studies have explored First Nations perspectives and tailoring primary care interventions to families and communities affected by methamphetamine use [7, 12]. The work by MacLean and colleagues also showed how historical trauma, socio‐economic marginalisation, stigma and shame detrimentally impact on the capacity of both First Nations communities families to deal with members' methamphetamine use [7, 12]. Similar to these findings, in this paper, study participants linked social and historic determinants—poverty, cultural loss, limited community resources, lack of appropriate services, discrimination and racism—as impeding factors on First Nations communities' and families' capacity to address methamphetamine use.

The role of culture in support for healing with Indigenous people has been noted in the treatment of alcohol and substance use in Australia, New Zealand, the United States and Canada [31, 32, 33, 34, 35]. For First Nations people in Australia, culture is recognised as a crucial element in the mental health and social support of [12, 21, 36, 37]. Original research in the United States highlights the requirement to culturally adapt clinical AOD treatments to affect greater acceptability and outcomes with Indigenous communities [38]. The review of culturally adapted psychological/psychosocial substance use interventions trialled in Australia and New Zealand found that all except one intervention showed significant improvements in at least one outcome domain [39]. The authors however conclude that overall the international evidence base is still inconclusive due to problematic study design [40, 41]. Similarly, the evidence of the impact of community‐based addiction recovery resources, such as mutual support groups with Indigenous people, are scarce [40, 41]. Continuous efforts are being made in clinical trials in the United States to improve study design and increase participation rates in research with Indigenous peoples [42].

Taking account of culture and strength‐based community‐embedded approaches—as sources of identity, healing and reducing the impact of stigma and shame for family supporters and in alcohol and other drug treatment—has vital practice and policy implications [12]. As previously identified [43], AOD services working from a holistic whole‐of‐life framework and culturally aware practice would mean integrating with other social support needs (housing, education, employment); organising group and recreational, cultural activities; prioritising local treatment options and outreach and recognising family networks as a complementary resource for professional services. A pilot study of an First Nations Wellbeing Intervention with workers in an First Nations community controlled health service, supporting families who experience problems related to methamphetamine use, found that the workers taking part in the intervention felt empowered and perceived it to be relevant to First Nations families [16].

Our paper highlights the adverse impacts of providing support for an individual who uses methamphetamine on First Nations family supporters. The multiple stressors on families are well documented in the international literature [29]. The scarcity of professional resources, social and peer support initiatives, and of evidence on how to support family members remains a concern [30]. Rane *et al*. [30, p. 1] note that ‘the field [of research] is in its infancy and needs urgent attention of researchers and policy makers’. A renewed interest in ecological factors—such as family and kinship, community, social support and culture—in reducing the harms of drug use and in the design and delivery of AOD treatment may provide a starting point for a shift in AOD practice and policy investment [36, 44].

Family has also gained recognition as a resource, in combination with professional services, in harm reduction and AOD treatment [45, 46, 47]. Connection with family, social and peer support groups and internet‐based groups, like with other chronic health conditions, have been found to help people to better manage their AOD use in the community compared with those who accessed standard care [48]. Acknowledging family, community and culture as resources in harm reduction and AOD treatment, in turn requires us to address families' support needs. This can be done through the development of culturally appropriate grassroot and professional interventions and services that support families' SEWB and build the capacity of families and extended family.

### 4.1 Limitations

We acknowledge that the value of integrating interview and focus group data is a topic of debate [49]. In this case, the decision was based on cultural and ethical considerations that we believe justify this decision. During analysis, we found that both interview and focus group data provided rich personal and community insights; therefore, the differences in the data collection processes did not pose a barrier to integrating the data at that point. Utilising the SEWB framework as an analytic tool proved both useful (in focusing on interpersonal relations, community or culture as central factors reducing harms and providing support) but also challenging due to the overlapping, integrated nature of issues and themes in emerged in our data.

The participants in our sample self‐nominated to take part and represent a select number of locations; therefore, our findings may not be representative of the experiences and concerns of people in other Aboriginal and Torres Strait Islander communities in Australia or Indigenous people in other countries.

## 5 CONCLUSION

Methamphetamine use and its harms to First Nations people, families and communities is a growing health and social concern for services, policy makers and communities themselves. To date, little research has explored these issues with First Nations community members and service providers. A central strength of our study is the large sample size and working in partnership with ACCHS and community researchers, as well as senior First Nations researchers on the team. Our paper adds to the understanding of the extensive support and care that family and extended family provide, in the context of scarce services and resources in regional and rural areas, where this study was carried out.

We found that historic and social determinants—racism, discrimination, stigma, fear of being judged—continue to affect service access for these populations; and there are limited culturally appropriate services and community support available to overcome these barriers. Efforts to support First Nations families and communities should be developed within the SEWB framework. The framework recognises wellbeing and healing as fundamentally connected to holistic health, kinship, community, culture and ancestry, and influenced by socioeconomic and historical determinants that shape First Nations peoples' everyday lives. The development and implementation of culturally appropriate interventions, recognising families' role in harm reduction and their own support needs, should be further investigated and trialled.

## CONFLICT OF INTEREST

The authors have no conflicts of interest.
